# In Vivo Safety and Efficacy of Chalcone-Loaded Microparticles with Modified Polymeric Matrix against Cutaneous Leishmaniasis

**DOI:** 10.3390/pharmaceutics15010051

**Published:** 2022-12-24

**Authors:** Ariane de J. Sousa-Batista, Natalia Arruda-Costa, Wallace Pacienza-Lima, Felipe Carvalho-Gondim, Rosiane F. Santos, Silvia A. G. Da-Silva, Maria Inês Ré, Bartira Rossi-Bergmann

**Affiliations:** 1 Institute of Biophysics Carlos Chagas Filho, Universidade Federal do Rio de Janeiro—UFRJ, Avenida Carlos Chagas Filho 373, Rio de Janeiro 21941-902, RJ, Brazil; 2 Nanotechnology Engineering Program, Alberto Luiz Coimbra Institute for Graduate Studies and Research in Engineering—COPPE, UFRJ, Avenida Horácio Macedo 2030, Rio de Janeiro 21941-972, RJ, Brazil; 3 Department of Microbiology, Immunology and Parasitology, Universidade do Estado do Rio de Janeiro—UERJ, Avenida Professor Manoel de Abreu 444, Rio de Janeiro 20550-170, RJ, Brazil; 4 RAPSODEE Center, IMT Mines Albi, CNRS, Jarlard Campus, University of Toulouse, CEDEX 09, F-81013 Albi, France

**Keywords:** microparticles, polyvinylpyrrolidone, chemotherapy, *Leishmania*

## Abstract

Current chemotherapy of cutaneous leishmaniasis (CL) is based on repeated systemic or intralesional administration of drugs that often cause severe toxicity. Previously, we demonstrated the therapeutic potential of biodegradable poly(lactic-co-glycolic acid) (PLGA) microparticles (MPs) loaded with 8% of the nitrochalcone CH8 (CH8/PLGA) prepared by a conventional bench method. Aiming at an industrially scalable process and increased drug loading, new MPs were prepared by spray drying: CH8/PDE with PLGA matrix and CH8/PVDE with PLGA + polyvinylpyrrolidone (PVP) matrix, both with narrower size distribution and higher drug loading (18%) than CH8/PLGA. Animal studies were conducted to evaluate their clinical feasibility. Both MP types induced transient local swelling and inflammation, peaking at 1–2 days, following a single intralesional injection. Different from CH8/PDE that released 90% of the drug in the ear tissue in 60 days, CH8/PVDE achieved that in 30 days. The therapeutic efficacy of a single intralesional injection was evaluated in BALB/c mice infected with *Leishmania (Leishmania) amazonensis* and golden hamsters infected with *L*. *(Viannia) braziliensis*. CH8/PVDE promoted greater reduction in parasite burden than CH8/PDE or CH8/PLGA, measured at one month and two months after the treatment. Thus, addition of PVP to PLGA MP matrix accelerates drug release in vivo and increases its therapeutic effect against CL.

## 1. Introduction

Leishmaniases are a complex of neglected tropical diseases (NTDs) caused by protozoans of the genus *Leishmania* that infect and multiply inside macrophages of the skin (cutaneous leishmaniasis—CL) or deep organs (visceral leishmaniasis). CL is the most common form of the disease and a serious public health problem affecting over 1 million people each year [1]. A great majority of CL patients present one to three localized ulcers, but in some cases parasites may spread and cause disfiguring diffuse and mucosal diseases. Currently, CL is treated with multiple parenteral or intralesional injections with pentavalent antimonials. Second-line drugs like pentamidine and liposomal amphotericin B are parenterally injected, and oral miltefosine is not a consensus treatment for CL. All these treatments have potential systemic toxicity and are given in repeated doses. They may be costly and susceptible to failure and resistance [2].

Ideally, CL treatment should be applied topically to avoid systemic adverse effects, but so far, no topical formulation has been able to penetrate the hypertrophied skin lesions and reach the parasites intracellularly in the dermis macrophages [3,4,5,6]. Intralesional injections with antimonials have circumvented the absorption issues [7], but repeated injections are still necessary due to rapid absorption of the highly hydrophilic drugs to the circulation, which may produce reported side effects, including anaphylactic shock [8].

**Figure 1:**
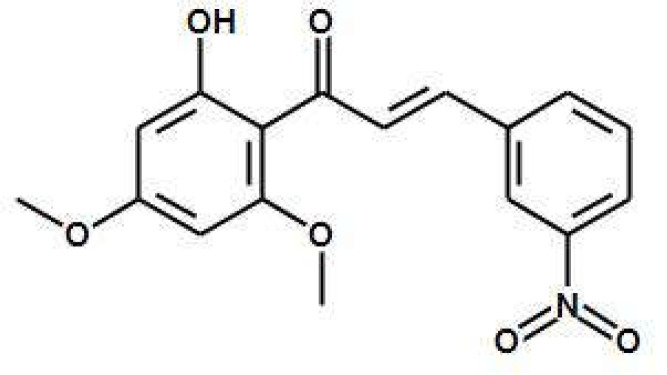
Chalcone CH8 (3-nitro-2-hydroxy-4,6-dimetoxychalcone).

Therefore, locally active drugs are strongly needed for CL. In this context, plant-derived chalcones have emerged as a promising drug candidate [9,10]. In addition to antibacterial, antifungal, anthelminthic, and immunomodulatory properties [11,12,13,14,15,16], chalcones have shown potent antileishmanial activity. Specifically, we have demonstrated the parasite target [17] and the broad spectrum of action of the synthetic 3-nitro-2′-hydro-4′,6′-dimethoxychalcone (CH8—Figure 1) against different *Leishmania* species in vitro, and its potent oral and intralesional efficacy in murine models of CL caused by *L. (Leishmania) amazonensis* and visceral leishmaniasis caused by *L. (Leishmania) infantum* [18,19].

Although local intralesional injections sound safer than parenteral or oral administrations, the need for repeated injections poses a logistical issue for patients living in remote areas or without access to health centers. A novel strategy developed by our group aimed at reducing the number of intralesional doses using sustained drug-delivery systems. For that, antileishmanial-loaded biodegradable microparticles (MPs) have been developed to form both a drug depot in the lesion tissue and serve as intracellular carriers after macrophage phagocytosis [20]. Poly(lactic-co-glycolic acid) (PLGA) is a biocompatible and biodegradable polymer widely approved by regulatory agencies such as the FDA (US), EMA (EC), and Anvisa (Brazil) for a range of clinical uses that include depot delivery systems (MPs) for treatment of chronic diseases, such as cancer and schizophrenia, that promote sustained i.m. or s.c. drug release over weeks or months [21]. Polymeric nanoparticles have also been experimentally employed to deliver chalcone or amphotericin B intracellularly in murine CL [22,23], but due to their smallness were not meant for sustained release, demanding repeated injections.

Recently, we described the production of CH8-loaded PLGA MPs, their effectiveness against intracellular parasites, and successful use in single-dose treatment of murine CL caused by *L. (Leishmania) amazonensis* [24]. In that study, the classical solvent precipitation and evaporation method (SPE) allowed a maximum of 8% drug loading. To optimize the CH8 MPs in terms of higher drug content and industrially scalable process, two new CH8-loaded MPs containing PLGA alone and PLGA plus polyvinylpyrrolidone—PVP as polymeric matrices and higher 18% CH8 loading were produced by spray drying [25]. Aiming to develop an effective, safe, local and single-dose treatment for CL with a new CH8 drug, in this study the safety, local drug kinetics and efficacy of these two MPs were evaluated in animals.

## 2. Materials and Methods

### 2.1. CH8 and Microparticles

The 3-nitro-2′-hydroxy-4′,6′-dimetoxychalcone (CH8) was synthesized by aldol condensation as described in [18]. CH8/PLGA was prepared by solvent precipitation and evaporation followed by lyophilization as described in [24]. CH8/PDE and CH8/PVDE differing in polymeric matrix were prepared using a Buchi B-290 spray dryer (Buchi Labortechnik AG, Flawil, Switzerland) equipped with Inert Loop B-295 and a two-fluid 2 mm nozzle. Drying conditions were: aspirator 85%; pump flow rate 360 mL/h and compressed nitrogen flow rate 500 L/h, inlet temperature 49 ± 1 °C. The resultant outlet temperature was 38 ± 2 °C. CH8/PDE was generated from PLGA (Purasorb PDLG 5004, 50:50, 0.83 dL/g; Corbion, Amsterdam, The Netherlands) alone, whereas CH8/PVDE was produced from a mixture of PVP K17 (Kollidon_17 PF endotoxin-free, BASF AG, Ludwigshafen, Germany) and PLGA of 1:10. The feeding solution was prepared by dissolving CH8 in a 1.5% (*w*/*v*) solution of PLGA or PLGA/PVP K17 in dichloromethane (Sigma-Aldrich, St. Louis, MO, USA) or in a mixture of ethyl acetate (Fluka, Buchs, Switzerland) in dichloromethane (1:2) (*v*/*v*). The drug:polymer mass proportion (dry basis) was fixed at 1:5. Blank PVDE MPs were prepared in the same way without CH8. All chemicals and solvents were of analytical or pharmaceutical grade, as described in [25].

### 2.2. Animals

BALB/c mice (female, 20 g, 8 weeks of age) were originally from Jackson Laboratory (Bar Harbor, ME, USA). Golden hamsters (*Mesocricetus auratus*, female, 80–90 g) were purchased from Fiocruz, Brazil. Animals were maintained under controlled temperature, filtered air and drinking water, autoclaved bedding, and commercial pelleted food. The experiments were approved by the committees for the ethics of animal use of the Federal University of Rio de Janeiro (mice, protocol CAUAP118) and Committee for the Ethics of Animal Use of Rio de Janeiro State University (hamsters, protocol CEUA/051/2017) in accordance with the Guide for the Care and Use of Laboratory Animals (NIH).

### 2.3. Mouse Ear Swelling Test

Induction of cutaneous hypersensitivity was evaluated using the mouse ear swelling test (MEST) [26]. Briefly, mice (*n* = 5/group) were presensitized by three s.c. injections in the rump with PBS (10 μL), free CH8, CH8/PDE or CH8/PVDE, all at 30 μg of CH8/10 μL of PBS on days −9, −7 and −5. This CH8 concentration was selected for the trials, as we had already proven its efficacy and safety in other studies [24]. On day 0, they were challenged in the ear in the same way. Positive controls received oxazolone solution (0.3% acetone) that was topically applied in both sites. Ear swelling was periodically measured with a digital micrometer (Mitutoyo) for up to 196 h. Sensitization is considered positive when above 10% of positive control.

### 2.4. Histopathology

Mice (*n* = 5/group) were injected s.c. in the ear pinnae with CH8, CH8/PDE or CH8/PVDE (all at 30 μg of CH8 in 10 μL of PBS). After 0 (untreated), 2 or 30 days, the animals were euthanized by isoflurane inhalation, and the ears were excised and fixed with 4% paraformaldehyde. Samples were dehydrated, embedded in paraffin wax, and 4-μm longitudinal slices were stained with Masson’s trichrome for light microscopy images (Nikon Eclipse—Ti).

### 2.5. In Vivo Drug Release

Mouse ears (*n* = 5/group) were injected with CH8, CH8/PDE or CH8/PVDE (all containing 30 μg of CH8) in 10 μL of PBS. At different time points, 5 animals/group were euthanized and their injected ears excised and individually ground in 1 mL of distilled water. The samples were lyophilized and the CH8 extracted with 1 mL of acetonitrile (Tedia) under ultrasound. After centrifugation, (10,000 rpm/10 min), CH8 was quantified in the supernatants by HPLC/UV (at 337 nm) as previously described [24].

### 2.6. In Vivo Efficacy against CL

*L. (Leishmania) amazonensis*-infected mice: BALB/c mice were infected in the ear with 2 × 10^6^ promastigotes of *L. amazonensis* (MHOM/BR/75/Josefa strain) transfected with green fluorescent protein (GFP) [27]. After 7 days of infection, the animals were treated with a single intralesional injection with CH8, CH8/PDE or CH8/PVDE (all at 30 μg of CH8 in 10 μL of PBS = 1.3 mg of CH8/Kg of body weight). Controls received 10 μL of PBS alone. On days 30 and 60 posttreatment, animals were euthanized by isoflurane inhalation, the ears removed from the base, ground in 1 mL of PBS and the fluorescence in the debris-free cell suspension measured by plate fluorimetry (435 nm excitation and 538 nm emission—FLx800, Bio-Tek Instruments, Inc., Winooski, VT, USA) for determination of *L. amazonensis* loads [28].

*L. (Viannia) braziliensis*-infected hamsters: Golden hamsters were infected in the ear with 10^5^ promastigotes of *L. braziliensis* (MCAN/BR/98/R619). Seven days later, the animals were given a single intralesional injection with CH8, CH8/PDE or CH8/PVDE (all at 120 μg of CH8 in 40 μL of PBS = 1.3 mg of CH8/Kg body weight). Controls received 40 μL of PBS alone or blank PVDE MPs. On day 60 posttreatment, animals were euthanized, and the infected ears were individually ground and assayed by limiting dilution assay (LDA) for determination of parasite loads [29].

### 2.7. Statistical Analysis

One-way ANOVA and the Tukey’s multiple comparison tests were performed. To compare the difference between two groups, the unpaired *t* test was used. All statistical analyses used GraphPad Prism 8 software.

## 3. Results

### 3.1. Spray Drying Allows Higher Drug Entrapment and Narrower Size Distribution

The characteristics of CH8-entrapped MPs were compared using SPE and spray-drying technologies. Table 1 shows that spray drying yielded MPs with higher CH8 loading (~18%) than SPE (~8%), regardless of added PVP. Mean particle sizes were similar (~6 to 8 μm), but spray drying allowed a more homogeneous size distribution (span < 2). Due to their better features, CH8/PDE and CH8/PVDE obtained by spray drying were herein elected for animal studies.

### 3.2. In Vivo Inflammation and Biodegradation of CH8/PDE and CH8/PDVE

**Figure 2:**
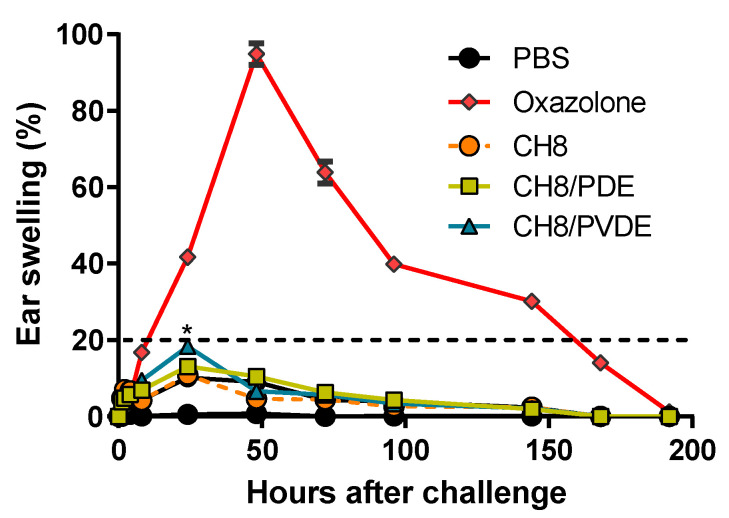
The mouse ear swelling test (MEST) following ear injection. Mice were given three s.c. injections in the rump with 10 μL of PBS or 30 μg of free CH8, CH8/PDE and CH8/PVDE on days −9, −7 and −5, and then challenged on day 0 with a homologous injection in the ear pinnae. Oxazolone (positive controls) was topically applied in the same sites (100%, 0.25 mm). Ear swelling was measured at the indicated times with a micrometer. The dotted line represents 20% of maximum oxazolone response. * *p* < 0.05 compared with PBS. Means ± SEM (*n* = 5).

The capacity of CH8/PDE and CH8/PVDE to induce local inflammation was evaluated by MEST and histopathology of treated ears. For MEST, presensitized mice were challenged in the ear pinnae with a single homologous s.c. injection, and ear thickness was measured for up to 196 h. It is worth mentioning that this is a maximized MEST where animals received three sensitizing injections, not topical applications in the rump. Figure 2 shows that CH8, CH8/PDE and CH8/PVDE induced a slight edema measured 24 h after challenge (12%, 15% and 19% of control oxazolone, respectively), waning afterwards.

**Figure 3:**
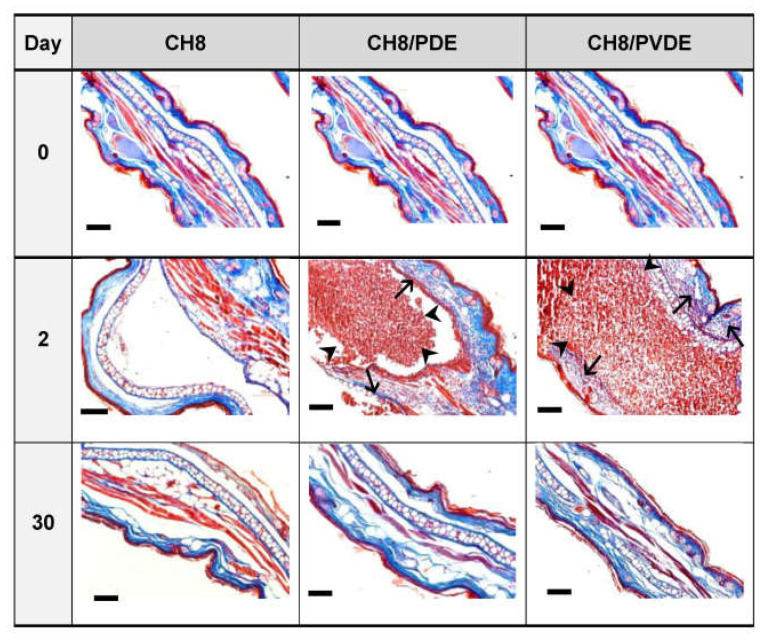
Histopathology and MP biodegradation in ear pinnae. Mice (*n* = 3) were injected in the ear pinnae with 10 μL of PBS containing 30 μg of CH8 in the free form, CH8/PDE or CH8/PVDE. On days 0 (untreated), 2 and 30, the ears were sliced and stained with Masson’s trichrome. Representative images are shown. MPs (arrowheads), stratum corneum, muscle fibers and the inflammatory infiltrate (arrows) are stained in red. Collagen and mucin are stained in blue. Bars = 100 μm.

For histopathology and biodegradation, a single s.c. injection with CH8 or MPs was given in the ear pinnae, and these were processed after 2 and 30 days. Figure 3 shows that after 2 days, abundant CH8/PDE and CH8/PVDE were visible at the injection site (arrowheads), as well as some inflammatory infiltrates (arrows), compatible with Figure 2. After 30 days, the MPs were unseen (biodegraded), inflammatory infiltrates had disappeared, and ear sizes had returned to normal.

### 3.3. CH8 Is More Rapidly Released from CH8/PVDE than CH8/PDE in the Ear

**Figure 4:**
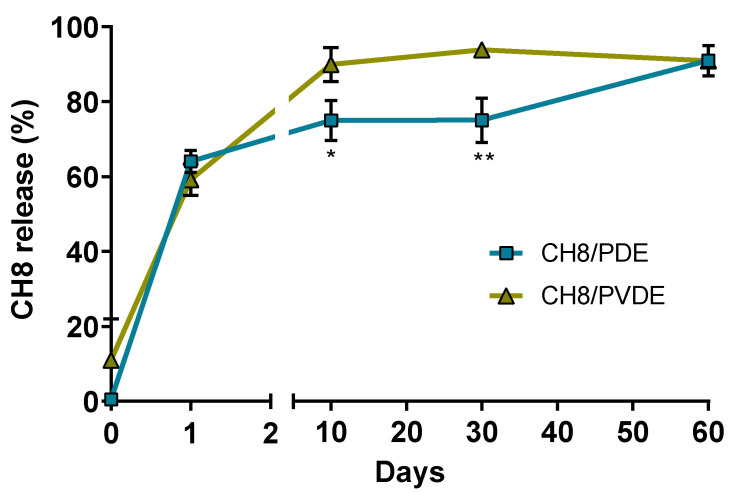
In vivo drug release. Mice were injected in the ear pinnae with 30 μg of CH8 loaded in either CH8/PDE or CH8/PVDE. At the indicated time points, mice were euthanized for quantification of CH8 in the ear tissue by HPLC-UV. Means ± SEM (*n* = 5). * *p* < 0.05, ** *p* < 0.01.

The amount of CH8 in the ear tissue was quantified at different times after a single injection with 30 μg of CH8 in each MP to evaluate the influence of the hydrophilic PVP polymer on drug-release speed. Figure 4 shows that 60% of CH8 was released within 24 h, irrespective of the formulation, while 90% release was achieved in 30 days with CH8/PVDE and 60 days with CH8-PLGA, indicating that PVP accelerates in vivo drug release.

### 3.4. Efficacy of a Single Dose of CH8/PVDE in Two Models of CL

**Figure 5:**
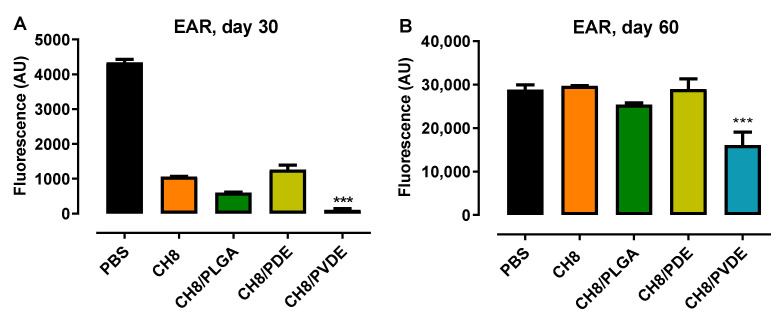
CH8/PVDE efficacy in *L. amazonensis*-infected mice. BALB/c mice were infected with 2 × 10^6^ *L. amazonensis*- GFP promastigotes in the ear pinnae and treated 7 days later with a single intralesional injection of CH8 (30 μg/dose—1.3 mg/kg body weight) in the free form or encapsulated in the indicated MPs. Controls received 10 μL of PBS alone. On days 30 and 60 posttreatment, mice were euthanized, and the parasite loads quantified by fluorimetry discounting the basal value of fluorescence of contralateral uninfected ears. A.U. = arbitrary units. Means ± SEM (*n* = 5) *** *p* < 0.001 against all treatments.

The efficacy of CH8/PVDE was evaluated in *L. amazonensis*-infected BALB/c mice and *L. braziliensis*-infected hamsters. In mice, efficacy was evaluated on days 30 and 60 postinfection (Figure 5). Due to transient inflammation, ear thicknesses were inconsistent with time; therefore, efficacy was scored as parasite burden. On day 30, all treatments had significantly reduced the parasite burden compared to PBS controls: free CH8 (86%), CH8/PLGA (71%) and CH8/PDVE (98%), respectively. CH8/PDVE was the only MP to be significantly (*p* < 0.001) more efficacious than CH8. On day 60, all treatments had lost the capacity to control parasite growth, except CH8/PDVE, which had 33% lower parasite burden than PBS.

**Figure 6:**
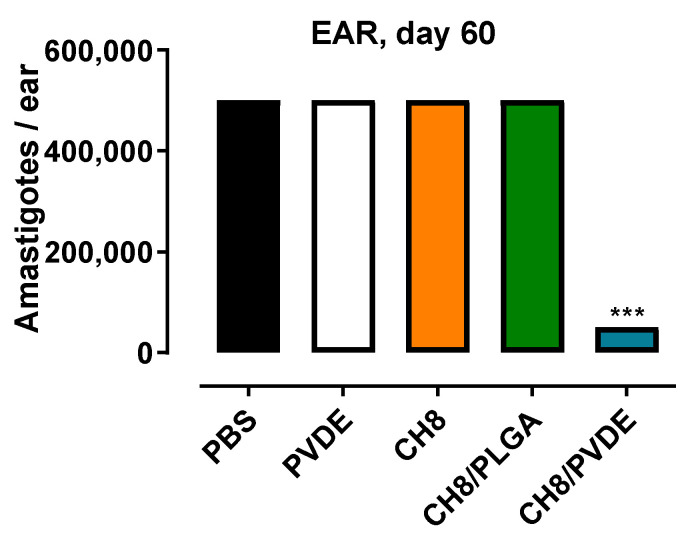
CH8/PVDE efficacy in *L. braziliensis*-infected hamsters. Golden hamsters were infected in the ear with 10^5^ promastigotes of *L. braziliensis*. After 7 days, the animals received a single intralesional injection of CH8 (120 μg/dose—1.3 mg/Kg body weight) encapsulated or not in the infected site. Controls received PBS alone or blank PVDE. On day 60 posttreatment, the parasite loads in the infected tissue were assayed by limiting dilution assay. Means ± SEM (*n* = 10) *** *p* < 0001 vs. all groups.

In hamsters infected with *L. braziliensis* for 60 days, CH8/PDVE was also the only CH8-loaded MP to control parasite growth by 90% in the ear site (Figure 6).

## 4. Discussion

The available chemotherapy for CL is extremely inadequate. Besides producing systemic toxicity, it requires repeated injections that are often difficult to administer to patients, particularly those living in remote forest areas, conflict zones, and where environmental disasters have occurred. In an attempt to develop new therapies for the most common CL localized form, we explored a new therapeutic strategy to promote not only localized but also single-dose treatment based on biodegradable MPs for sustained drug release allied with intracellular drug discharge. Previously, we produced and characterized CH8-loaded microparticles prepared by solvent evaporation [24] and spray drying [25].

Further spray-drying modifications led to CH8/PDE and CH8/PVDE MPs with higher CH8 content (18%) than previously obtained with the solvent evaporation process (8%). The skin safety and in situ degradation and drug release of those new MPs were evaluated here prior to proof of concept in animals with CL. Both MP types proved to be acceptably safe in terms of cutaneous hypersensitivity induction and local inflammation at the injection site. The edema produced was not strong enough (<20% of topical oxazolone), transient and totally reversed in few days, with no sign of inflammatory infiltrates after 30 days of injection. Such proinflammatory features of CH8/PDE and CH8/PVDE MPs are compatible with those described for other PLGA MPs [30,31], and have been associated with the PLGA conversion of macrophages to the proinflammatory (M1) polarization state [32]. In turn, M1 macrophages are associated with increased intracellular parasite killing and CL infection control [33]. Thus, besides promoting sustained drug release and intracellular drug delivery, PLGA systems can also benefit CL chemotherapy in terms of immunomodulatory adjuvanticity.

The drug-release kinetics at the injection site indicated that both CH8/PDE and CH8/PVDE MPs presented a biphasic curve, as expected for PLGA MPs [34], with an initial burst release in the first 24 h followed by a slower release up to 60 days. CH8/PVDE showed a faster release profile than CH8/PLGA, compatible with other studies demonstrating accelerated drug release from nanoparticles and other MPs when PLGA is blended with PVP [35,36]. This effect can be explained by the higher PVP dissolution in contact with an aqueous medium in relation to PLGA, leading to the formation of pores in the MPs [37,38] that allow the water to get into the MPs, resulting in faster drug release [39].

To verify how faster drug release promoted by PVP could impact CL treatment, MPs efficacy was compared using two rodent models of infection with different immunological responses against *Leishmania* [40]. Although CL follow-up is often done by measuring lesion growth, we opted for omitting the curves here because of the interference of local inflammation with the ear thickness. Thus, as in VL assessments, only the more relevant parasite burden was expressed. In the BALB/c mouse model of infection with *L. amazonensis*, all CH8 MPs successfully controlled the parasite burden, measured 30 days after treatment. Amongst them, CH8/PVDE showed the greatest efficacy (98%) compared to CH8/PLGA (86%) and CH8/PDE (71%), denoting the importance of PVP, possibly due to its faster drug release. In this sense, it is conceivable that the faster polymer-degradation products have contributed to increased macrophage activation and hence parasite control [41]. The observation that after 60 days of treatment, only CH8/PVDE was still able to reduce the parasite loads (33%) indicates that a new dose boost may be necessary.

The most active MPs tested against *L. amazonensis* were chosen to be tested against *L. braziliensis* infection, the most prevalent *Leishmania* species in the Americas. Hamsters were chosen for their high susceptibility and chronicity of infection with *L. braziliensis* that best resemble the human disease [42,43]. A single time point was chosen (60 days) due to the large number of animals needed. Empty PVDE was without effect, confirming our previous findings on the lack of efficacy of blank PLGA MPs against CL [24,44]. As late as 60 days, only CH8/PVDE was controlling parasite growth in hamsters, when the numbers of *L. braziliensis* parasites were 90% smaller than controls. It is possible that as in mice, the other CH8 treatments were effective at earlier times (e.g., day 30) but not afterwards. Although no sterile cure was achieved, clinical cure of American CL is rarely associated with absence of parasites, with the remaining parasites being important to sustain memory immune responses [45]. Thus, the results achieved with a single dose can still be considered very significant.

## 5. Conclusions

In this study, we showed that a new chalcone CH8 formulation comprised of PLGA added to PVP (CH8/PVDE) can be successfully produced by the industrially scalable method of spray drying to facilitate pharmaceutic manufacturing. More importantly, different from the previous MPs made of sole PLGA matrix (CH8/PLGA) whose antileishmanial effect decreases after 30 days, the new PVDE spray-dried formulation made with PLGA-PVP polymeric blend accelerates in situ drug release and at the same time allows the effect of a single intralesional injection to last for at least 60 days. This is a great achievement in terms of CL treatment, which will not only promote patient adherence but also reduce treatment costs.

## Figures and Tables

**Table 1:** Microparticle characteristics.

Microparticle	Method	Polymer	Solvent	% CH8	Size (μm) D (4,3)	Span
CH8/PLGA	SPE	PLGA	DCM	7.8 ± 1.5	6.2	2.4
CH8/PDE	Spray drying	PLGA	DCM:EA	18.1 ± 0.0	6.4	1.8
CH8/PVDE	Spray drying	PLGA:PVP	DCM:EA	18.1 ± 0.1	7.9	1.7

SPE = solvent precipitation and evaporation; PVP = polyvinylpyrrolidone K17; DCM = dichloromethane; EA = ethyl acetate.

## Data Availability

Not applicable.
